# Beyond Inhibition: A Dual-Process Perspective to Renew the Exploration of Binge Drinking

**DOI:** 10.3389/fnhum.2014.00405

**Published:** 2014-06-04

**Authors:** Séverine Lannoy, Joël Billieux, Pierre Maurage

**Affiliations:** ^1^Laboratory for Experimental Psychopathology, Psychological Sciences Research Institute, Université Catholique de Louvain, Louvain-la-Neuve, Belgium

**Keywords:** binge drinking, alcohol-dependence, inhibition, emotion, dual-process

## Abstract

Binge drinking is a widespread alcohol-consumption pattern in youth and is linked to cognitive consequences, mostly for executive functions. However, other crucial factors remain less explored in binge drinking and notably the emotional-automatic processes. Dual-process model postulates that addictive disorders are not only due to impaired reflective system (involved in deliberate behaviors), but rather to an imbalance between under-activated reflective system and over-activated affective-automatic one (involved in impulsive behaviors). This proposal has been confirmed in alcohol-dependence, but has not been tested in binge drinking. The observation of comparable impairments in binge drinking and alcohol-dependence led to the “continuum hypothesis,” suggesting similar deficits across different alcohol-related disorders. In this perspective, applying the dual-process model to binge drinking might renew the understanding of this continuum hypothesis. A three-axes research agenda will be proposed, exploring: (1) the affective-automatic system in binge drinking; (2) the systems’ interactions and imbalance in binge drinking; (3) the evolution of this imbalance in the transition between binge drinking and alcohol-dependence.

## Introduction

Alcohol abuse and dependence constitute a central public health concern (Nutt et al., 2010) and are directly involved in more than 4% of all death worldwide (Rehm et al., 2009). The cognitive and cerebral consequences of alcohol-dependence have been extensively described (Bühler and Mann, 2011; Stavro et al., 2013). However, as the huge majority of these studies focused on prolonged alcohol-dependence, other types of alcohol-consumption patterns have been far less explored. This is particularly true for binge drinking, characterized by the repeated alternations between intense intoxications and repeated withdrawals. This consumption habit is now a widespread phenomenon in youth, as 40–60% of adolescents and young adults in Western countries are now considered as binge drinkers (Archie et al., 2012; Johnston et al., 2012). Nevertheless, while binge drinking is far more frequent than alcohol-dependence in youth (Hingson et al., 2006), its cognitive and cerebral consequences have only been explored during the last decade (Hermens et al., 2013). It has been shown that binge drinking is associated with rapid alterations in attention, memory, and executive functions, and with functional brain impairments, mostly in frontal regions (Schweinsburg et al., 2010; Campanella et al., 2013; Jacobus and Tapert, 2013). These recent data led to the proposal that binge drinking is centrally associated with high-level cognitive functions impairments and can thus be described as a disorder of the reflective system responsible for controlled behaviors.

While deficits in executive functions and inhibition are indeed a central feature in binge drinking, the exclusive focus on these processes hampered the exploration of other crucial factors. Recent influential model of alcohol-related disorders, namely the dual-process model (Noël et al., 2010; Wiers et al., 2013), postulates that alcohol-dependence is not only associated with impaired executive control, but rather with a simultaneous reduction of executive control and an increased impact of automatic and emotional processes. The links between binge drinking and alcohol-dependence are still to be clarified, but the observation of parallel impairments among binge drinkers and alcohol-dependent individuals (e.g., Maurage et al., 2012) and the observation that binge drinking during adolescence increases the risk of alcohol-dependence in adulthood (Bonomo et al., 2004) led some authors to propose a connection between binge drinking and alcohol-dependence, summarized in the continuum hypothesis (Enoch, 2006): binge drinkers would display qualitatively similar but quantitatively less marked impairments than alcohol-dependent individuals. Accordingly, binge drinking might thus also be associated with hyper-activation of this affective-automatic system underlying the uncontrolled and emotional behaviors. Nevertheless, the integrity of this system in binge drinking has not been experimentally tested. More globally, if the dual-process model is now well-validated in alcohol-dependence, it has not been directly tested in earlier stages of alcohol-related problems, and particularly in binge drinking. The main aim of this paper is thus to underline the need to test the validity of the dual-process model in binge drinking in order to have a better understanding of this phenomenon and to directly explore the continuum hypothesis. After having described the theoretical framework of this model and its validity for alcohol-dependence, a literature review of the data available concerning impairments in executive control among binge drinkers will be proposed, before reporting the few evidences concerning the deficits for automatic and emotional processes. We will then propose a research agenda centrally focused on three experimental axes: (1) the specific evaluation of affective-automatic system in binge drinking; (2) the elucidation of the interactions between reflective and affective-automatic systems and their imbalance in binge drinking; (3) the building of a developmental perspective, i.e., the exploration of the evolution of this imbalance across the successive stages of alcohol-dependence, from early abuse in binge drinking to chronic misuse in alcohol-dependence.

## Dual-Process Model in Alcohol-Dependence

Dual-process model (Mukherjee, 2010) posits that decision-making abilities result from the interactions between two systems: (1) the “reflective system,” involved in the cognitive evaluation of the stimuli by means of memory and executive functions, responsible for controlled-deliberate responses; (2) the “affective-automatic system,” involved in the emotional evaluation of the stimuli, initiating automatic-appetitive responses. Neuroscience studies (Daw et al., 2005; Hampton and O’Doherty, 2007) sustained these proposals by showing that these systems are associated with distinct cerebral networks, i.e., limbic network for the affective-automatic system and prefrontal network for the reflective system.

Alcohol-dependence is characterized by short-term decision-making (e.g., consuming to relieve a negative emotional state) leading to the persistence of maladjusted behaviors despite their long-term negative consequences. Following the dual-process view, recent models (Noël et al., 2010; Wiers et al., 2013) proposed that alcohol-dependence relies on an impairment of the two systems, leading to an imbalance. First, the affective-automatic system is over-activated by emotional or alcohol-related stimuli, leading to impulsive prepotent behaviors. Second, the reflective system is impaired, leading to an inability to voluntarily inhibit the consumption. These models have been supported by empirical data showing: (1) emotional impairments in alcohol-dependence (i.e., reduced performance in the identification of the emotional content of faces, voices, postures, or words), and particularly an over-reactivity of the limbic system when confronted with socio-affective (Uekermann et al., 2005; Maurage et al., 2008) or alcohol-related stimuli (Bjork et al., 2008); (2) marked alterations of prefrontal abilities, mostly in tasks requiring executive control, inhibition (Stavro et al., 2013), or high-level deliberative processes (Coskunpinar et al., 2013).

Dual-process model has thus been largely validated in alcohol-dependence and constitutes a reliable theoretical framework. However, this model has received very little support from studies exploring other alcohol-consumption patterns and it is thus unknown whether it is also valid for other alcohol-related problems, and particularly for binge drinking. Indeed, the reflective system has been tested among binge drinkers by exploring executive functions, but the alterations of the affective-automatic system and the potential imbalance between systems remain to be explored. The scarcity of the data exploring the systems related to dual-process model thus currently hampers to generalize this model to binge drinking.

## Reflective System in Binge Drinking

The cognitive and brain impairments related to the reflective system have been extensively explored (Hermens et al., 2013; Petit et al., 2014) in binge drinking among adolescents (12–18 years) and young adults (18–25 years). Neuropsychological studies focusing on behavioral tasks have highlighted the rapid appearing of cognitive consequences among binge drinkers compared to abstinent matched controls. Indeed, after only 1 year of binge drinking, impairments have been identified in perceptive-motor (Brumback et al., 2007) and attentional abilities (Zeigler et al., 2005). Several studies have also shown altered higher-level functions, notably working and episodic memory (Scaife and Duka, 2009). However, as executive impairments are the crucial feature of alcohol-dependence, studies mostly explored these deficits in binge drinking, consistently showing impairments for planning, updating, flexibility, or inhibition (Hartley et al., 2004; Tapert et al., 2004; Goudriaan et al., 2007).

Importantly, these behavioral results have been extended by neuroscience data. Electrophysiological studies first showed that the event-related potentials associated with attentional and executive processes have abnormal latency and amplitude during working memory tasks in binge drinking (Ehlers et al., 2007; Crego et al., 2012; Lopez-Caneda et al., 2013). Moreover, neuroimaging studies suggested the brain areas involved: anatomical studies showed reduced cortical thickness among male binge drinkers (Squeglia et al., 2012) and reduced activations in prefrontal areas during memory and executive tasks were observed (Schweinsburg et al., 2010, 2011; Xiao et al., 2013). However, the relationship between binge drinking and cognitive-cerebral deficits is still under debate. Several longitudinal studies in adolescents and young adults showed a progressive appearance of the deficits with alcohol-consumption increase (Goudriaan et al., 2007; Maurage et al., 2009; Jacobus et al., 2013; Mota et al., 2013), but others identified executive deficits before any alcohol consumption (Nigg et al., 2004; Weafer and Fillmore, 2008), suggesting that reduced fronto-parietal activations in children might predict binge drinking (Norman et al., 2011).

The available data thus clearly suggest that binge drinking is associated with important impairments of the reflective system. However, the scarcity of longitudinal studies and the absence of direct comparison between binge drinkers and alcohol-dependent individuals prevent the elaboration of a developmental perspective on the reflective system’s deficits across the successive stages of alcohol-related problems, and the validity of the continuum hypothesis is still to be tested.

## Affective-Automatic System in Binge Drinking

Affective-automatic system has been extensively explored in alcohol-dependence. On the one hand, alcohol-dependent individuals present higher automatic processing and attentional biases toward alcohol-related stimuli (Field and Cox, 2008; Field et al., 2009). As this increased sensitivity to alcohol-related cues plays a role in the persistence of craving and increases the relapse risk after detoxification, interventions developed to reduce this sensitivity and thus lower consumption have flourished (Fadardi and Cox, 2009; Schoenmakers and Wiers, 2010). On the other hand, alterations in the processing of emotional and interpersonal stimuli have been observed in alcohol-dependent individuals, these patients notably displaying over-interpretation of negative emotional signals (Kornreich et al., 2001; Maurage et al., 2008) and increased sensitivity to social rejection (Maurage et al., 2012). Alcohol-dependence is thus associated with over-reactivity of the affective-automatic system, but a systematic exploration of this system is lacking in binge drinking.

Concerning the increased automatic approach for alcohol-related cues, a study among young adults (Field et al., 2008) suggested that an increased sensitivity toward alcohol cues during early alcohol consumption might be involved in the evolution toward alcohol-dependence. This proposal has received several confirmations among heavy drinkers or social drinkers, by showing that these alcohol-consumption patterns are associated with stronger craving (Grüsser et al., 2006) and with over-sensitivity to alcohol, as measured in various implicit tasks (Wiers et al., 2005; Field et al., 2008; Peeters et al., 2012; Kessler et al., 2013; Petit et al., 2013). Interestingly, recent longitudinal studies showed that the intensity of automatic approach tendency (particularly when combined with poor inhibition) significantly predicts future alcohol consumption among adolescents, confirming the importance of the automatic system in the initiation of excessive alcohol consumption (Thush and Wiers, 2007; Peeters et al., 2013). These studies clarified the involvement of the automatic system in alcohol consumption, but nearly all of them included various alcohol-consumption patterns without specifically exploring binge drinking. Actually, only one study (Thush and Wiers, 2007) focused on binge drinking in adolescence, but did not compare binge drinking with other consumption patterns. It thus remains unknown whether the impairments described for the automatic system in youth are modulated by a specific binge drinking consumption or are present whatever the excessive alcohol consumption is (e.g., heavy drinking, hazardous drinking). This question is important as it has been shown (Maurage et al., 2012) that the repeated alternation of intoxication and withdrawal found in binge drinking leads to different consequences than other alcohol consumption patterns, even when the total amount of alcohol consumed per week is identical. Binge drinking thus seems to constitute a particularly harmful habit, but this proposal is still to be explored for the automatic system.

Concerning the emotional disturbances, very little has been done in binge drinking. Several studies have described increased depression, anxiety, or negative mood levels in young adults binge drinkers for self-reported measures (Townshend and Duka, 2005; Bekman et al., 2013), but these results have not been confirmed by experimental studies focusing on mood induction or affective perturbations. Only two studies directly explored the processing of emotional stimuli in binge drinking, by means of affective prosody decoding tasks. The first one (Maurage et al., 2009), conducted among young adults, showed that after only 9 months of binge drinking, the electrophysiological components associated with the processing of emotional human voices are disrupted. The second one (Maurage et al., 2013) extended these results by showing that young adults binge drinkers have a reduced performance in emotional voice categorization, this behavioral impairment being linked with a double brain alteration: binge drinkers presented reduced activation in the voice processing area (i.e., superior temporal gyrus), but showed increased activation in another area usually not involved in emotional processing (i.e., middle frontal gyrus). This result can be interpreted as reflecting a compensatory activation (i.e., the recruitment of preserved brain areas to compensate for impaired activity in areas usually involved in the task), as observed earlier for working memory abilities (Schweinsburg et al., 2010; Campanella et al., 2013). This preliminary evidence suggests that binge drinking might be associated with impairments of the affective system, but further studies should confirm and extend these data.

## Research Program: Applying the Dual-Process Perspective to Binge Drinking

The application of dual-process model in alcohol-dependence led to an in-depth renewal of this field, but its validity has not been explored in binge drinking. Indeed, several theoretical models of binge drinking have been proposed (e.g., Oei and Morawska, 2004; Elliott and Ainsworth, 2012), but they were focused on the psychological variables predicting binge drinking and did not take into account the cognitive and cerebral consequences of this drinking habit, and the influence of these deficits on the evolution of alcohol consumption. Moreover, as shown above, the impairments related to the affective-automatic system are still to be explored. Two major limits currently hamper to obtain a reliable theoretical background to apply dual-process model to binge drinking: first, the interactions between reflective and affective-automatic systems are unknown, and second no study directly compared the systems’ deficits in binge drinking and alcohol-dependence. In view of these current limitations, three main research axes will now be proposed to end up in the emergence of a new model of binge drinking, capitalizing on the dual-process perspective. These three axes will combine cross-sectional (Axes 1 and 2) and longitudinal (Axis 3) designs to respectively explore:

(1) *The affective-automatic system in binge drinking*: as state above, the affective correlates of binge drinking remain nearly unexplored. A first part of this axis will thus be to offer a precise exploration of the automatic system over-activation, by proposing a multi-evaluation using various validated tasks (e.g., implicit association, memory association, attentional bias) to assess the impairments presented by binge drinkers as compared to non-drinkers. A second part will be to explore the emotional system in binge drinking. Indeed, future studies should test whether these processes are already impaired in adolescents and young adults binge drinkers by means of a comprehensive exploration ranging from basic emotional abilities (e.g., facial or vocal emotion decoding) to complex affective skills (e.g., empathy, emotional intelligence) and interpersonal aptitudes (e.g., social integration and interactions). As emotional impairments have been found to play a crucial role in the development and maintenance of alcohol-dependence, understanding the early alterations of this affective system and its implications in the first stages of alcohol-dependence is required.
(2) *The interactions between systems and their imbalance in binge drinking*: the main proposal of the dual-process model is that addictions result from an imbalance between reflective and affective-automatic systems. This proposal remains to be confirmed in binge drinking. It has been shown that the combination of hypo-activated reflective system and hyper-activated affective-automatic one might favor the development of excessive alcohol consumption in adolescence (Peeters et al., 2013), but the direct interactions between these systems have not been tested in experimental settings. Importantly, it has been proposed that impulsive and risk-taking behaviors in adolescence might be due to the differential maturation of the two systems, the reflective system being undeveloped while the affective-automatic one would be over-activated (Steinberg, 2007). Neuroimaging studies in healthy adolescents supported this hypothesis (e.g., Gogtay et al., 2004; Blakemore et al., 2007), but the presence of an imbalance in risk-taking youth has not been proven. As binge drinking is a very frequent risk-taking habit, it constitutes an ideal population to test this proposal. A direct exploration of the systems’ imbalance should thus be conducted in this population, not only by exploring each system separately, but rather by means of experimental tasks specifically testing emotion–cognition interactions (e.g., emotional flanker task, prepotent response inhibition tasks using affective stimuli). This paradigm shift from correlational approach (i.e., separately exploring the two systems) to experimental one is required to actually test the main proposal related to dual-process model. Finally, adding a group of alcohol-dependent individuals in these cross-sectional designs would allow the comparison of these systems’ imbalance across binge drinking and alcohol-dependence. This would offer new insights concerning the validity of the continuum hypothesis, stating that binge drinking and alcohol-dependence would not constitute separate entities but would rather be the two successive stages of a single phenomenon (Enoch, 2006). However, the in-depth exploration of this hypothesis can only be done by longitudinal approaches, as proposed in the following axis.
(3) *The evolution of this imbalance across the successive stages of alcohol-dependence*: while several studies have supported the continuum hypothesis by showing that binge drinkers display similar impairments than alcohol-dependent individuals regarding cognitive and emotional abilities, but also at the cerebral level (Maurage et al., 2009, 2012; Sanhueza et al., 2011), no study directly explored this transition between binge drinking and alcohol-dependence within a cohort of participants. This third research axis will use a longitudinal approach with multiple testing sessions starting in early adolescence to explore the mutual influences between binge drinking habits and systems’ impairments. This longitudinal perspective will bring two main insights as compared to cross-sectional one: first, by starting before the onset of alcohol consumption, it will clarify the existence of pre-existing cognitive-emotional deficits in binge drinking. Second, as this exploration will be carried on through adolescence and adulthood, it will explore the evolution of the deficits during the modification of drinking habits (i.e., the potential reduction of these deficits when binge drinking stops, and conversely their progression when binge drinking evolves toward alcohol-dependence). To our knowledge, this would constitute the first direct exploration of the transition between binge drinking and alcohol-dependence, allowing to determine the crucial predictors of this transition.

To conclude, our proposal is that an in-depth exploration of the dual-process model in binge drinking might totally renew this model by adding a developmental perspective, leading to strong implications. At the theoretical level, it would notably renew the continuum hypothesis by extending it toward affective-automatic system’s deficits and systems’ interactions. At the clinical level, it might lead to the creation of prophylactic rehabilitation programs proposing early interventions to re-equilibrate the balance between reflective and affective-automatic systems at early stages of excessive alcohol consumption.

## Conflict of Interest Statement

The authors declare that the research was conducted in the absence of any commercial or financial relationships that could be construed as a potential conflict of interest.

## References

[B1] Archie S. Zangeneh Kazemi A. Akhtar-Danesh N. (2012). Concurrent binge drinking and depression among Canadian youth: prevalence, patterns, and suicidality. Alcohol 46, 165–172 10.1016/j.alcohol.2011.07.001 21824740

[B2] Bekman N. M. Winward J. L. Lau L. L. Wagner C. C. Brown S. A. (2013). The impact of adolescent binge drinking and sustained abstinence on affective state. Alcohol. Clin. Exp. Res. 37, 1432–1439 10.1111/acer.12096 23550712 PMC3706493

[B3] Bjork J. M. Smith A. R. Hommer D. W. (2008). Striatal sensitivity to reward deliveries and omissions in substance dependent patients. Neuroimage 42, 1609–1621 10.1016/j.neuroimage.2008.06.035 18672069 PMC2615386

[B4] Blakemore S.-J. den Ouden H. Choudhury S. Frith C. (2007). Adolescent development of the neural circuitry for thinking about intentions. Soc. Cogn. Affect. Neurosci. 2, 130–139 10.1093/scan/nsm009 17710201 PMC1948845

[B5] Bonomo Y. A. Bowes G. Coffey C. Carlin J. B. Patton G. C. (2004). Teenage drinking and the onset of alcohol dependence: a cohort study over seven years. Addiction 99, 1520–1528 10.1111/j.1360-0443.2004.00846.x 15585043

[B6] Brumback T. Cao D. King A. (2007). Effects of alcohol on psychomotor performance and perceived impairment in heavy binge social drinkers. Drug Alcohol Depend. 91, 10–17 10.1016/j.drugalcdep.2007.04.013 17560739 PMC2764986

[B7] Bühler M. Mann K. (2011). Alcohol and the human brain: a systematic review of different neuroimaging methods. Alcohol. Clin. Exp. Res. 35, 1771–1793 10.1111/j.1530-0277.2011.01540.x 21777260

[B8] Campanella S. Peigneux P. Petit G. Lallemand F. Saeremans M. Noël X. (2013). Increased cortical activity in binge drinkers during working memory task: a preliminary assessment through a functional magnetic resonance imaging study. PLoS ONE 8:e62260 10.1371/journal.pone.0062260 23638017 PMC3636085

[B9] Coskunpinar A. Dir A. L. Cyders M. A. (2013). Multidimensionality in impulsivity and alcohol use: a meta-analysis using the UPPS model of impulsivity. Alcohol. Clin. Exp. Res. 37, 1441–1450 10.1111/acer.12131 23578176 PMC3732812

[B10] Crego A. Cadaveira F. Parada M. Corral M. Caamaño-Isorna F. Rodríguez Holguín S. (2012). Increased amplitude of P3 event-related potential in young binge drinkers. Alcohol 46, 415–425 10.1016/j.alcohol.2011.10.002 22459872

[B11] Daw N. D. Niv Y. Dayan P. (2005). Uncertainty-based competition between prefrontal and dorsolateral striatal systems for behavioral control. Nat. Neurosci. 8, 1704–1711 10.1038/nn1560 16286932

[B12] Ehlers C. L. Phillips E. Finnerman G. Gilder D. Lau P. Criado J. (2007). P3 components and adolescent binge drinking in Southwest California Indians. Neurotoxicol. Teratol. 29, 153–163 10.1016/j.ntt.2006.11.013 17196788

[B13] Elliott M. A. Ainsworth K. (2012). Predicting university undergraduates’ binge-drinking behavior: a comparative test of the one- and two-component theories of planned behavior. Addict. Behav. 37, 92–101 10.1016/j.addbeh.2011.09.005 21945010

[B14] Enoch M. (2006). Genetic and environmental influences on the development of alcoholism. Ann. N Y Acad. Sci. 1094, 193–201 10.1196/annals.1376.019 17347351

[B15] Fadardi J. S. Cox W. M. (2009). Reversing the sequence: reducing alcohol consumption by overcoming alcohol attentional bias. Drug Alcohol Depend. 101, 137–145 10.1016/j.drugalcdep.2008.11.015 19193499

[B16] Field M. Cox W. (2008). Attentional bias in addictive behaviors: a review of its development, causes, and consequences. Drug Alcohol Depend. 97, 1–20 10.1016/j.drugalcdep.2008.03.030 18479844

[B17] Field M. Kiernan A. Eastwood B. Child R. (2008). Rapid approach responses to alcohol cues in heavy drinkers. J. Behav. Ther. Exp. Psychiatry 39, 209–218 10.1016/j.jbtep.2007.06.001 17640615

[B18] Field M. Munafò M. R. Franken I. H. A. (2009). A meta-analytic investigation of the relationship between attentional bias and subjective craving in substance abuse. Psychol. Bull. 135, 589–607 10.1037/a0015843 19586163 PMC2999821

[B19] Gogtay N. Giedd J. Lusk L. Hayashi K. Greenstein D. Vaituzis A. (2004). Dynamic mapping of human cortical development during childhood through early adulthood. Proc. Natl. Acad. Sci. U.S.A. 101, 8174–8179 10.1073/pnas.0402680101 15148381 PMC419576

[B20] Goudriaan A. E. Grekin E. R. Sher K. J. (2007). Decision making and binge drinking: a longitudinal study. Alcohol. Clin. Exp. Res. 31, 928–938 10.1111/j.1530-0277.2007.00378.x 17403069 PMC2667377

[B21] Grüsser S. M. Mörsen C.P. Flor H. (2006). Alcohol craving in problem and occasional alcohol drinkers. Alcohol Alcohol. 41, 421–425 10.1093/alcalc/agl035 16636008

[B22] Hampton A. N. O’Doherty J. P. (2007). Decoding the neural substrates of reward-related decision making with functional MRI. Proc. Natl. Acad. Sci. U.S.A. 104, 1377–1382 10.1073/pnas.0606297104 17227855 PMC1783089

[B23] Hartley D. E. Elsabagh S. File S. E. (2004). Binge drinking and sex: effects on mood and cognitive function in healthy young volunteers. Pharmacol. Biochem. Behav. 78, 611–619 10.1016/j.pbb.2004.04.027 15251270

[B24] Hermens D. F. Lagopoulos J. Tobias-Webb J. De Regt T. Dore G. Juckes L. (2013). Pathways to alcohol-induced brain impairment in young people: a review. Cortex 49, 3–17 10.1016/j.cortex.2012.05.021 22789780

[B25] Hingson R. W. Heeren T. Winter M. R. (2006). Age at drinking onset and alcohol dependence: age at onset, duration, and severity. Arch. Pediatr. Adolesc. Med. 160, 739–746 10.1001/archpedi.160.7.739 16818840

[B26] Jacobus J. Squeglia L. M. Bava S. Tapert S. F. (2013). White matter characterization of adolescent binge drinking with and without co-occurring marijuana use: a 3-year investigation. Psychiatry Res. 214, 374–381 10.1016/j.pscychresns.2013.07.014 24139957 PMC3900025

[B27] Jacobus J. Tapert S. F. (2013). Neurotoxic effects of alcohol in adolescence. Annu. Rev. Clin. Psychol. 9, 703–721 10.1146/annurev-clinpsy-050212-185610 23245341 PMC3873326

[B28] Johnston L. O’Malley P. M. Bachman J. G. Schulenberg J. E. (2012). Monitoring the Future National Survey Results on Drug Use, 1975–2011: Volume II, College Students and Adults Ages 19–50, 1st Edn Ann Arbor: Institute for Social Research, The University of Michigan

[B29] Kessler K. Pajak K. M. Harkin B. Jones B. (2013). A working memory bias for alcohol-related stimuli depends on drinking score. Psychol. Addict. Behav. 27, 23–31 10.1037/a0028664 22642857

[B30] Kornreich C. Blairy S. Philippot P. Dan B. Foisy M.-L. Hess U. (2001). Impaired emotional facial expression recognition in alcoholism compared with obsessive-compulsive disorder and normal controls. Psychiatry Res. 102, 235–248 10.1016/S0165-1781(01)00261-X 11440774

[B31] Lopez-Caneda E. Cadaveira F. Crego A. Doallo S. Corral M. Gomez-Suarez A. (2013). Effects of a persistent binge drinking pattern of alcohol consumption in young people: a follow-up study using event-related potentials. Alcohol Alcohol. 48, 464–471 10.1093/alcalc/agt046 23695975

[B32] Maurage P. Bestelmeyer P. E. G. Rouger J. Charest I. Belin P. (2013). Binge drinking influences the cerebral processing of vocal affective bursts in young adults. Neuroimage Clin. 3, 218–225 10.1016/j.nicl.2013.08.010 24179866 PMC3791281

[B33] Maurage P. Campanella S. Philippot P. Martin S. de Timary P. (2008). Face processing in chronic alcoholism: a specific deficit for emotional features. Alcohol. Clin. Exp. Res. 32, 600–606 10.1111/j.1530-0277.2007.00611.x 18241315

[B34] Maurage P. Joassin F. Philippot P. Heeren A. Vermeulen N. Mahau P. (2012). Disrupted regulation of social exclusion in alcohol-dependence: an fMRI study. Neuropsychopharmacology 37, 2067–2075 10.1038/npp.2012.54 22510722 PMC3398714

[B35] Maurage P. Pesenti M. Philippot P. Joassin F. Campanella S. (2009). Latent deleterious effects of binge drinking over a short period of time revealed only by electrophysiological measures. J. Psychiatry Neurosci. 34, 111–118 19270761 PMC2647570

[B36] Mota N. Parada M. Crego A. Doallo S. Caamaño-Isorna F. Rodríguez Holguín S. (2013). Binge drinking trajectory and neuropsychological functioning among university students: a longitudinal study. Drug Alcohol Depend. 133, 108–114 10.1016/j.drugalcdep.2013.05.024 23791027

[B37] Mukherjee K. (2010). A dual system model of preferences under risk. Psychol. Rev. 117, 243–255 10.1037/a0017884 20063971

[B38] Nigg J. T. Glass J. M. Wong M. M. Poon E. Jester J. M. Fitzgerald H. E. (2004). Neuropsychological executive functioning in children at elevated risk for alcoholism: findings in early adolescence. J. Abnorm. Psychol. 113, 302–314 10.1037/0021-843X.113.2.302 15122950

[B39] Noël X. Bechara A. Brevers D. Verbanck P. Campanella S. (2010). Alcoholism and the loss of willpower: a neurocognitive perspective. J. Psychophysiol. 24, 240–248 10.1027/0269-8803/a000037 21765575 PMC3136191

[B40] Norman A. L. Pulido C. Squeglia L. M. Spadoni A. D. Paulus M. P. Tapert S. F. (2011). Neural activation during inhibition predicts initiation of substance use in adolescence. Drug Alcohol Depend. 119, 216–223 10.1016/j.drugalcdep.2011.06.019 21782354 PMC3208054

[B41] Nutt D. J. King L. A. Phillips L. D. (2010). Drug harms in the UK: a multicriteria decision analysis. Lancet 376, 1558–1565 10.1016/S0140-6736(10)61462-6 21036393

[B42] Oei T. P. S. Morawska A. (2004). A cognitive model of binge drinking: the influence of alcohol expectancies and drinking refusal self-efficacy. Addict. Behav. 29, 159–179 10.1016/S0306-4603(03)00076-5 14667427

[B43] Peeters M. Monshouwer K. van de Schoot R. A. Janssen T. Vollebergh W. A. Wiers R. W. (2013). Automatic processes and the drinking behavior in early adolescence: a prospective study. Alcohol Clin. Exp. Res 37, 1737–1744 10.1111/acer.12156 23763261

[B44] Peeters M. Wiers R. W. Monshouwer K. van de Schoot R. Janssen T. Vollebergh W. A. M. (2012). Automatic processes in at-risk adolescents: the role of alcohol-approach tendencies and response inhibition in drinking behavior: alcohol use in at-risk adolescents. Addiction 107, 1939–1946 10.1111/j.1360-0443.2012.03948.x 22632107

[B45] Petit G. Kornreich C. Verbanck P. Campanella S. (2013). Gender differences in reactivity to alcohol cues in binge drinkers: a preliminary assessment of eventrelated potentials. Psychiatry Res. 209, 494–503 10.1016/j.psychres.2013.04.005 23684055

[B46] Petit G. Maurage P. Kornreich C. Verbanck P. Campanella S. (2014). Binge drinking in adolescents: A review of neurophysiological and neuroimaging research. Alcohol Alcohol. 49, 198–206 10.1093/alcalc/agt172 24302160

[B47] Rehm J. Mathers C. Popova S. Thavorncharoensap M. Teerawattananon Y. Patra J. (2009). Global burden of disease and injury and economic cost attributable to alcohol use and alcohol-use disorders. Lancet 373, 2223–2233 10.1016/S0140-6736(09)60746-7 19560604

[B48] Sanhueza C. García-Moreno L. M. Expósito J. (2011). Weekend alcoholism in youth and neurocognitive aging. Psicothema 23, 209–214 21504671

[B49] Scaife J. C. Duka T. (2009). Behavioural measures of frontal lobe function in a population of young social drinkers with binge drinking pattern. Pharmacol. Biochem. Behav. 93, 354–362 10.1016/j.pbb.2009.05.015 19497334

[B50] Schoenmakers T. M. Wiers R. W. (2010). Craving and attentional bias respond differently to alcohol priming: a field study in the pub. Eur. Addict. Res. 16, 9–16 10.1159/000253859 19887804

[B51] Schweinsburg A. D. McQueeny T. Nagel B. J. Eyler L. T. Tapert S. F. (2010). A preliminary study of functional magnetic resonance imaging response during verbal encoding among adolescent binge drinkers. Alcohol 44, 111–117 10.1016/j.alcohol.2009.09.032 20113879 PMC2845466

[B52] Schweinsburg A. D. Schweinsburg B. C. Nagel B. J. Eyler L. T. Tapert S. F. (2011). Neural correlates of verbal learning in adolescent alcohol and marijuana users: fMRI in adolescent users. Addiction 106, 564–573 10.1111/j.1360-0443.2010.03197.x 21134014 PMC3423457

[B53] Squeglia L. M. Sorg S. F. Schweinsburg A. D. Wetherill R. R. Pulido C. Tapert S. F. (2012). Binge drinking differentially affects adolescent male and female brain morphometry. Psychopharmacology 220, 529–539 10.1007/s00213-011-2500-4 21952669 PMC3527131

[B54] Stavro K. Pelletier J. Potvin S. (2013). Widespread and sustained cognitive deficits in alcoholism: a meta-analysis: alcoholism and cognition. Addict. Biol. 18, 203–213 10.1111/j.1369-1600.2011.00418.x 22264351

[B55] Steinberg L. (2007). Risk taking in adolescence: new perspectives from brain and behavioral science. Curr. Dir. Psychol. Sci. 16, 55–59 10.1111/j.1467-8721.2007.00475.x

[B56] Tapert S. F. Schweinsburg A. D. Barlett V. C. Brown S. A. Frank L. R. Brown G. G. (2004). Blood oxygen level dependent response and spatial working memory in adolescents with alcohol use disorders. Alcohol. Clin. Exp. Res. 28, 1577–1586 10.1097/01.ALC.0000141812.81234.A6 15597092

[B57] Thush C. Wiers R. W. (2007). Explicit and implicit alcohol-related cognitions and the prediction of future drinking in adolescents. Addict. Behav. 32, 1367–1383 10.1016/j.addbeh.2006.09.011 17125932

[B58] Townshend J. M. Duka T. (2005). Binge drinking, cognitive performance and mood in a population of young social drinkers. Alcohol. Clin. Exp. Res. 29, 317–325 10.1097/01.ALC.0000156453.05028.F5 15770105

[B59] Uekermann J. Daum I. Schlebusch P. Trenckmann U. (2005). Processing of affective stimuli in alcoholism. Cortex 41, 189–194 10.1016/S0010-9452(08)70893-1 15714901

[B60] Weafer J. Fillmore M. T. (2008). Individual differences in acute alcohol impairment of inhibitory control predict ad libitum alcohol consumption. Psychopharmacology 201, 315–324 10.1007/s00213-008-1284-7 18758758 PMC4310478

[B61] Wiers R. W. Gladwin T. E. Hofmann W. Salemink E. Ridderinkhof K. R. (2013). Cognitive bias modification and cognitive control training in addiction and related psychopathology: mechanisms, clinical perspectives, and ways forward. Clin. Psychol. Sci. 1, 192–212 10.1177/2167702612466547

[B62] Wiers R. W. van de Luitgaarden J. van den Wildenberg E. Smulders F. T. Y. (2005). Challenging implicit and explicit alcohol-related cognitions in young heavy drinkers. Addiction 100, 806–819 10.1111/j.1360-0443.2005.01064.x 15918811

[B63] Xiao L. Bechara A. Gong Q. Huang X. Li X. Xue G. (2013). Abnormal affective decision making revealed in adolescent binge drinkers using a functional magnetic resonance imaging study. Psychol. Addict. Behav. 27, 443–454 10.1037/a0027892 22486330

[B64] Zeigler D. W. Wang C. C. Yoast R. A. Dickinson B. D. McCaffree M. A. Robinowitz C. B. (2005). The neurocognitive effects of alcohol on adolescents and college students. Prev. Med. 40, 23–32 10.1016/j.ypmed.2004.04.044 15530577

